# Carbon Nanoparticles’ Impact on Processability and Physical Properties of Epoxy Resins—A Comprehensive Study Covering Rheological, Electrical, Thermo-Mechanical, and Fracture Properties (Mode I and II)

**DOI:** 10.3390/polym11020231

**Published:** 2019-02-01

**Authors:** Hauke Meeuw, Johann Körbelin, Valea Kim Wisniewski, Ali Shaygan Nia, Adrián Romaní Vázquez, Martin Rudolf Lohe, Xinliang Feng, Bodo Fiedler

**Affiliations:** 1 Institute of Polymer and Composites, Hamburg University of Technology (TUHH), Denickestr. 15, 20173 Hamburg, Germany; johann.koerbelin@tuhh.de (J.K.); valea.wisniewski@tuhh.de (V.K.W.); fiedler@tuhh.de (B.F.); 2 Chair for Molecular Functional Materials and Center for Advancing Electronics Dresden (cfaed), Technische Universität Dresden, Mommsenstraße 4, 01069 Dresden, Germany; ali.shaygan_nia@mailbox.tu-dresden.de (A.S.N.); adrian.romani_vazquez@mailbox.tu-dresden.de (A.R.V.); martin.lohe@tu-dresden.de (M.R.L.); xinliang.feng@tu-dresden.de (X.F.)

**Keywords:** nano composites, viscosity, percolation, fracture toughness, mode I, mode II

## Abstract

A trade-off between enhancement of physical properties of the final part and the processability during manufacturing always exists for the application of nanocarbon materials in thermoset-based composites. For different epoxy resins, this study elaborates the impact of nanocarbon particle type, functionalization, and filler loading on the resulting properties, i.e., rheological, electrical, thermo-mechanical, as well as the fracture toughness in mode I and mode II loading. Therefore, a comprehensive set of carbon nanoparticles, consisting of carbon black (CB), single-walled carbon nanotubes (SWCNT), multi-walled carbon nanotubes (MWCNT), few layer graphene (FLG), and electrochemically expanded graphite (ExG), in purified or functionalized configuration was introduced in various epoxy resins, with different molecular weight distributions. A novel technique to introduce sharp cracks into single-edge notched bending (SENB) fracture toughness specimens led to true values. SWCNT show highest potential for increasing electrical properties without an increase in viscosity. Functionalized MWCNT and planar particles significantly increase the fracture toughness in mode I by a factor of two.

## 1. Introduction

Epoxy resins offer high-performance properties, namely light weight potential, improvement of corrosion resistance, barrier properties, fire retardancy, and electrical conductivity [1], for a wide range of adhesive applications, such as matrices in fiber composites or coatings [2]. However, due to their high grade of cross-linking they possess low fracture toughness and therefore, a brittle nature [3]. It is widely reported that this can be overcome by addition of various types of nanoparticles [4,5]. They promise high potential for fracture toughness improvement without affecting other mechanical properties. This occurs due to improved energy dissipation and internal stress re-allocation. The quality of dispersion is crucial, because the nanoparticles eventually act as defects in case of poor dispersion [6,7]. Addition of carbon-based nanoparticles leads to an electrical conductivity of the otherwise isolating polymer. The electrical conductivity can be predicted regarding particle concentration, their aspect ratio and waviness [8]. By variation of particle type and filler content the electrical conductivity can be precisely adjusted [9,10]. Carbon nanoparticles typically lead to an enormous increase of the nanocomposite’s viscosity in dependency of filler type and loading [11,12]. The viscosity is a critical parameter regarding the processability of polymers. Reddy et al. observed that fillers migrate into the free volume and increase it. This leads to a non-favorable decrease of nanocomposite’s glass transition temperature [13]. Nadiv et al. investigated the optimal concentration of nanoparticle filler loading regarding fracture toughness in mode I and correlated it with the electrical and rheological percolation [14,15]. They concluded that the rheological percolation occurs prior to electrical percolation and optimal nanoparticle concentration lays slightly below the loading leading to rheological percolation. Domun et al. reported this optimal filler content regarding the mode I fracture toughness increase for a wide set of different type of nanoparticles in their review article as well [5]. Numerous other groups underpinned this observation [5,16,17,18,19,20,21]. The fact that the electrical percolation requires a higher filler loading than the rheological percolation leads to a trade-off between the enhancement of physical properties and processability of such nanocomposites. The effect of carbon nanoparticles on mode II fracture toughness is discussed controversially in literature. Various groups report an increase of mode II fracture toughness [19,20,22,23]. A continuous decrease is reported by Moghadam et. al. [24] and Shadlou et al. [22]. Zappalorto et al. observed a maximum in mode I and mode II fracture toughness by variation of filler content [21]. This paper aims to investigate the influence of carbon nanoparticles such as carbon black (CB), single-walled carbon nanotubes (SWCNT), multi-walled carbon nanotubes (MWCNT), few-layer graphene (FLG), and electrochemically expanded graphite (ExG) on the composite’s viscosity, electrical conductivity, Young’s modulus, glass transition temperature (T_G_) and the fracture toughness in mode I and II of epoxy resins.

## 2. Materials and Methods

### 2.1. Epoxy Resins

All used epoxy resins base on bisphenol-A-diglycidyl-ether (DGEBA). Epikote 162 offers the lowest viscosity, which increases with Epikote 827, 828LVEL and 828, respectively. Curing agent was a mixture of Epikure RIMH 137 and RIMH 134 (80/20 by weight). These are a commonly used curing agents in the wind-craft energy industry. According to its data sheet, RIMH 137 consists of poly(oxypropylene)diamine (50–75 wt.%) and 3-aminomethyl-3,5,5-trimethylcyclohexylamine (35–50 wt.%). The ingredients of RIMH 134 are 2-piperazin-1-ylethylamine (35–50 wt.%), 3-aminomethyl-3,5,5-trimethylcyclohexylamine (25–35 wt.%), poly (oxypropylene) diamine (20–25 wt.%), benzyl alcohol (3–7 wt.%), phenol(4,4′-(1-methylethylidene)bis-polymer with 5-amino-1,3,3-trimethylcyclohexanemethan amine and (chloromethyl)oxirane (1–2.5 wt.%) and branched 4-nonylphenol (0.25–0.5 wt.%). Hexion, Germany, supplied all resins and curing agents. Table 1 lists the properties of the neat resins according to their data sheets.

### 2.2. Carbon Nanoparticles

An extensive set of particle types was chosen to evaluate the effect of different carbon nanoparticle morphologies on the physical properties. Commercially available carbon black Printex L purchased from Orion Engineered Carbons, Luxembourg, offers a spherical shape. It is a carbon black pigment recommended for conductive coating applications. Its structure makes it easy to disperse, because it cannot entangle and the size distribution is narrow. MWCNT NC7000, supplied by Nanocyl, Belgium, are cylindrical and rigid tubes. Additionally, research grade MWCNT, supplied by Future Carbon, Germany, were used. CNTB are the reference type of the neat MWCNT. CNTA and CNTP are argon and atmospheric plasma treated CNTB, respectively. CNTN are CNTB type MWCNT equipped with elastomer side chains, which are amino-functionalized and therefore able to crosslink with the epoxy resins [25]. SWCNT Tuball 75, supplied by OCSiAl, Luxembourg, are cylindrical and, due to their small diameter, flexible tubes. They possess a highly entangled and bundled structure. OCSiAl provided a purified variant of their SWCNT, too. FLG Avan2, supplied by Avanzare Innovación Tecnológica S.L., Spain, has a planar structure. A second evaluated planar particle type was electrochemically expanded graphite. The manufacturing was performed according to the process described by Parvez et al. [26] at Technische Universität Dresden, which produces the ExG under a license from Sixonia Tech GmbH, Germany. For compounding, the ExG was first separated from the electrolyte and washed with deionized water. Instead of a subsequent dispersion step, which is typically employed to destroy the graphene agglomerates and liberate pristine exfoliated graphene sheets, the expanded, but still agglomerated product, was transferred into ethanol, to prepare highly concentrated slurries. Table 2 gives the properties of the used nanoparticles.

Specific surface areas were determined by a Surfer Gas Adsorption Porosimeter, Thermo Scientific, USA, using nitrogen. Scanning electron microscopy (SEM) was performed on a Zeiss Supra VP 55, Zeiss, Germany, with an acceleration voltage between 5 and 10 kV to analyze the morphologies of the particles. During SEM energy dispersive X-ray spectroscopy (EDX) was performed, too. Additionally, the usage of transmission electron microscopy (TEM) Talos F200X, Thermo Scientific, USA, revealed the number of walls and bundle sizes. An acceleration voltage of 200 kV was used. Carbon particles were ultrasonicated in methanol, placed on a TEM grid and dried at 80 °C. TEM and SEM images are shown in Appendix A. The impurity content was also determined with thermogravimetric analyses (TGA) with a TGA/DSC 1—thermogravimetric analyzer from Mettler-Toledo, USA. Samples were placed in a 70 µL alumina crucible covered with a pierced lid. Temperature cycle started with a heat up from room temperature to 50 °C under nitrogen atmosphere. Afterwards an isothermal step at 50 °C for 10 min in nitrogen atmosphere followed to keep the sample in steady state. The sample was heated under oxygen atmosphere to 950 °C, was held at this temperature for another 10 min and then cooled down to room temperature. Heating rates were 10 K/min and cooling rate 30 K/min.

### 2.3. Dispersion

The nanoparticles were incorporated into the neat epoxy systems via a seven-step three-roll milling process, using an 80E Plus, Exakt Advanced Technologies, Germany. Rollers have a diameter of 80 mm and are made from steel. A detailed description of the three-roll milling process can be found in [27,28]. Table 3 lists the used parameters.

### 2.4. Rheology

Addition of curing agent took place after dispersion. The viscosity of the composites was determined directly after addition of curing agent and subsequent mixing. A rheometer ARES RDA-III 28 from TA Instruments, USA, was used for this purpose. Strain-sweeps were performed from 0.1 to 100% at a frequency of 5 Hz. The test setup was a plate-plate configuration with a radius *R* of 40 mm and a spacing *h* of 500 µm. This gap spacing ensures that big agglomerates are not trapped between both plates. The procedure was adapted from a previous study [29].

### 2.5. Electrical Conductivity

For plate manufacturing, the materials were infused into a closed mold under vacuum. The curing cycle was chosen according to data sheet, with 24 h at room temperature and 15 h at 80 °C. After demolding, the samples were milled from the plate into the desired geometry. Prior to sawing and notching for fracture toughness sample preparation, the flanks of the manufactured rectangular specimens were covered with silver paint for determination of electrical conductivity. The electrical DC resistance of the cured rectangular specimens was measured at operating voltage of 1 V between the silver paint covered flanks with a Keithley 2601A system source meter, Keithley, USA.

### 2.6. Dynamic Mechanical Thermal Analysis

For dynamical-thermal-mechanical-analysis (DMTA) specimens with a thickness of 2 mm were prepared from the manufactured plates. DMTA was performed with a Gabo Eplexor 500N, NETZSCH GABO Instruments, Germany. The heating rate was set to 3 K/min, clamping distance 30 mm and test frequency 5 Hz. The temperature range was 20 to 180 °C.

### 2.7. Fracture Toughness in Mode I and II

**Figure 1:**
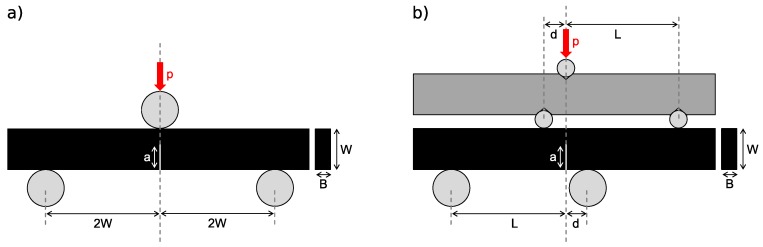
Test setup and definitions for determination of (**a**) $K_{IC}$ in a three-point-bending test (**b**) $K_{IIC}$ in asymmetric four-point-bending test adapted from [31].

The critical value of the crack front stress intensity factor characterizes the resistance of a material against failure by fracture. Figure 1a shows the test setup for the determination of critical stress intensity factor $K_{IC}$ in a three-point-bending test (3PBT). The pre-notched rectangular sample is loaded under bending, resulting in a pure tension loading of the crack tip (mode I). For a critical stress value, the crack starts to proceed. This results in failure of the material, for brittle materials such as glassy polymers. The determination of the mode II critical stress intensity factor $K_{IIC}$ requires a crack tip loading under nearly pure in-plane shear. Fett suggests a realization in an asymmetric four-point-bending test (4PBT) of a pre-notched sample, shown in Figure 1b [30]. He analyzed the crack tip loading and failure with finite element modeling and energy considerations.

**Figure 2:**
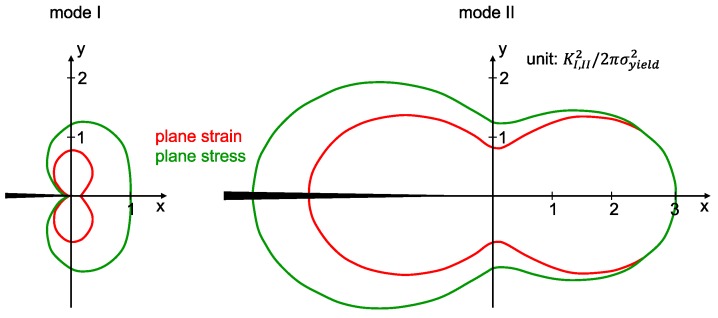
Dimensions of the plastic zones for (**left**) mode I and (**right**) mode II crack tip loading adapted from [32,33].

The geometry of the specimen is given by the thickness *W*, distance from support to crack *L*, crack length *a* and asymmetric distance *d*. Figure 2 gives the dimensions of the plastic zones in mode I and II for the condition $K_{IC}=K_{IIC}$, respectively.

The determination of stress distributions along the specimen ($\sigma_{x}\left(x,y\right)$, $\tau_{xy}\left(x,y\right)$) leads to numerical descriptions of those stresses with endless sum terms. To obtain a reasonable accuracy for the stress distribution, the sums are cut after a certain order of terms. The stress distribution in combination with the geometry of the specimens leads to an integrational description of the stress intensity factors:

(1) $$K_{I}={\int_{0}}^{a}h_{I}\left(\eta /a,a/W\right)\tau_{xy}\left(\eta \right)d\eta$$

and

(2) $$K_{II}={\int_{0}}^{a}h_{II}\left(\eta /a,a/W\right)\sigma_{x}\left(\eta \right)d\eta$$

with η=(W/2)−y.

Considering a boundary-collocation technique it is possible to find formulas for the weight functions $h_{I}$ and $h_{II}$:

(3) $$h_{I}=\sqrt{\frac{2}{\pi a}}\frac{1}{\sqrt{1-\eta /a}{\left(1-a/W\right)}^{3/2})}\left[{\left(1-\frac{a}{W}\right)}^{3/2}+\sum A_{\alpha \beta }{\left(1-\eta /a\right)}^{\alpha +1}{\left(\frac{a}{W}\right)}^{\beta }\right]$$

(4) $$h_{II}=\sqrt{\frac{2}{\pi a}}\frac{1}{\sqrt{1-\eta /a}{\left(1-a/W\right)}^{1/2}}\left[{\left(1-\frac{a}{W}\right)}^{1/2}+\sum A_{\alpha \beta }{\left(1-\eta /a\right)}^{\alpha +1}{\left(\frac{a}{W}\right)}^{\beta }\right]$$

with tabulated coefficients for $A_{\alpha \beta }$. Integrating over the length of the crack the stress intensity factors can be calculated after following equation:

(5) $$K_{I}=\frac{P}{WB}\left(1-\frac{d}{L}\right)\sqrt{\pi a}F_{I}$$

(6) $$K_{II}=\frac{P}{WB}\left(1-\frac{d}{L}\right)\sqrt{\pi a}F_{II}$$

The geometric factors $F_{I}$ and $F_{II}$ can be obtained from tables according to the specimen’s geometry or can be calculated following the equations stated below:

(7) $$F_{II}=3.9204\xi -5.1295\xi^{2}+14.4766L\xi^{3}-26.2916\xi^{4}+17.073\xi^{5}$$

with ξ=a/W.

Important ratios to determine the mode II fracture toughness (in-plane shear) in an asymmetric 4PBT are following: The length to width ratio should be

(8) $$\frac{L}{W}=2.5,$$

so that the distance between supports has a minimized influence on the result.

Other geometry ratios are

(9) $$0.4<\frac{a}{W}<0.6$$

so that the influence of $K_{I}$ becomes small. Ayatollahi et al. investigated different ratios by finite element modeling and concluded that 0.5 is an appropriate value to choose, because it secures the maximum distance to both, the bottom and top edges [19].

(10) $$0.35<\frac{d}{W}<0.625$$

leads to an independent factor $K_{II}$ from the ratio d/W.

By choosing following ratios for a given material thickness of W=4.5 mm

(11) $$\frac{L}{W}=2.5,\;\frac{a}{W}=0.5,\;\frac{d}{W}=0.5$$

following relations can be derived

(12) $$w=\frac{L}{2.5}=2a\Rightarrow L=5a=5d$$

The mode II fracture toughness (crack tip loading in in-plane shear) specimen must be of a minimum length to secure the asymmetric bending test configuration without gliding through the supports:

$$L_{total}=2L+\Delta L=50\;\mathrm{mm}$$

According to this geometry with the ratios stated above the geometry factors are the following: $F_{I}=0.0806$ and $F_{II}=1.3661$, so that the stress intensity factors are:

(13) $$K_{I}=\frac{P}{WB}\left(1-\frac{d}{L}\right)\sqrt{\pi a}\cdot 0.0806$$

(14) $$K_{II}=\frac{P}{WB}\left(1-\frac{d}{L}\right)\sqrt{\pi a}\cdot 1.3661$$

Since mode II is investigated in the asymmetric 4PBT and mode I in 3PBT, only the equation for $K_{II}$ must be considered. The used geometry is listed in Table 4.

Therefore, the lower and upper support span length for asymmetric 4PBT to introduce the load is

L+d=19.5mm+3.9mm=23.4mm

The mode I fracture toughness (crack tip loading in tension) is determined in a symmetrical three-point bending test. Important geometrical ratios for the sample are specified in ASTM D5045-14 [34] with:

(15) $$2<\frac{W}{B}<4$$

and

(16) $$0.45<\frac{a}{W}<0.55.$$

The mode I fracture toughness in 3PBT can be calculated by the following equation after ASTM D5045-14 [34]:

(17) $$K_{I}=\frac{P}{B\sqrt{W}}f\left(x\right)$$

with x=a/W and

(18) $$f\left(x\right)=6x^{1/2}\frac{1.99-x\left(1-x\right)\left(2.15-3.93x+2.7x^{2}\right)}{\left(1+2x\right){\left(1-x\right)}^{3/2}}$$

The geometry of the specimen is the same as for the mode II test, only the lower support span and the load introduction differ, refer to Table 5.

The total crack length *a* consists of a sawing cut and a sharp pre-notch. This sharp pre-notch should be at least twice as deep as the sawing cuts width to ensure a negligible notch-effect. For the used 150 μm diamond sawing blade, the pre-notch must be at least 300 μm deep. To ensure this boundary condition the sharp notch is chosen to be $a_{blade}=500$ μm. Therefore, the sawing cut has to be

$$a_{saw}=3.9\;\mathrm{mm}-0.5\;\mathrm{mm}=3.4\;\mathrm{mm}$$

deep, to reach the total crack length a=3.9 mm.

**Figure 3:**
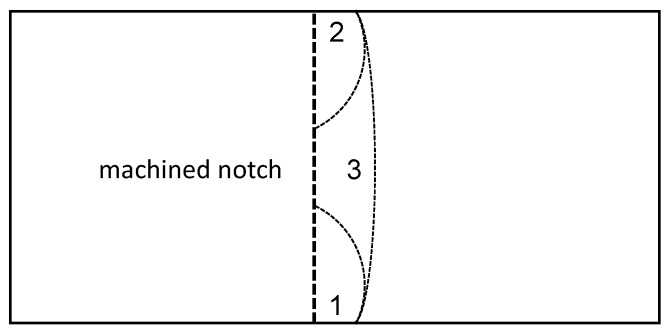
Visualization of novel notching technique for pre-crack insertion. 1, 2 and 3 denote the sequence of crack insertion.

The effect of different notching techniques for glassy polymeric SENB samples have been intensively investigated in literature [35,36,37,38]. They all conclude that an infinitesimal sharp notch leads to a homogeneous stress field and no prior plastic deformations in front of the crack tip. To ensure this, a three-step notching procedure was used as described in Figure 3. The crack consists of three unifying crack fronts. The first two fronts are inserted with a fresh razor blade from the base edges of the machined notch. These cracks stop to propagate at a certain point due to increasing cross-section in front of the crack tip. It remains a triangular cross-section in the middle of the machined notch base. In this the third crack front is initiated and the crack fronts form one very sharp notch with a defined length.

The 3PBT and asymmetric 4PBT were conducted with a Zwick/Roell Z2.5 universal testing machine, Zwick/Roell, Germany, with a cross-head speed of 10 mm/min. The diameter of the support rollers was 6 mm. 3PBT was performed according to ASTM D5045-14 [34] and the asymmetric 4PBT was performed according to the setup of Fett [30]. After testing, the pre-crack length was measured with an optical light microscope Olympus BX-51, Olympus, Germany. The averaged value was determined from three measuring points. A Phenom XL, Thermo Scientific, USA, was used to capture SEM images for crack initiation investigations. The operating voltage was 5 kV and the detector Topo A was used. Edges of the sample were coated with silver paint to improve the charge dissipation.

## 3. Results and Discussion

This section will give the results of the experiments beginning with the dependency of composite viscosity on particle type and filler loading. After that the electrical properties are discussed followed by a presentation of the thermo-mechanical and fracture toughness properties.

### 3.1. Rheology

**Figure 4:**
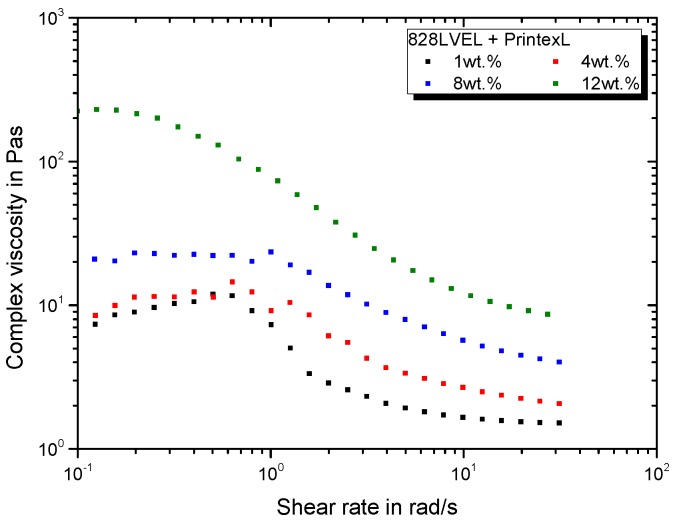
Rheological behavior of CB modified 828LVEL and RIMH137/134.

The rheological behavior, primarily determined by the viscosity, is one of the main properties to consider for the manufacturing of parts out of polymers and their composites. Besides the knowledge of the material’s viscosity, the knowledge of the rheological behavior in dependency of applied shear rate is important. Figure 4 gives the rate dependent complex viscosity for CB modified epoxy resin with addition of the curing agent. Until a specific shear rate, the complex viscosity exhibits a plateau. Reaching a critical shear rate, the material shows a shear-thinning behavior. A substantial increase in viscosity is observed for CB at high concentrations (12 wt.%), whereas the impact is low up to a loading of 8 wt.%. The critical shear rate for beginning of shear-thinning is strongly decreased for a filler loading 12 wt.%.

**Figure 5:**
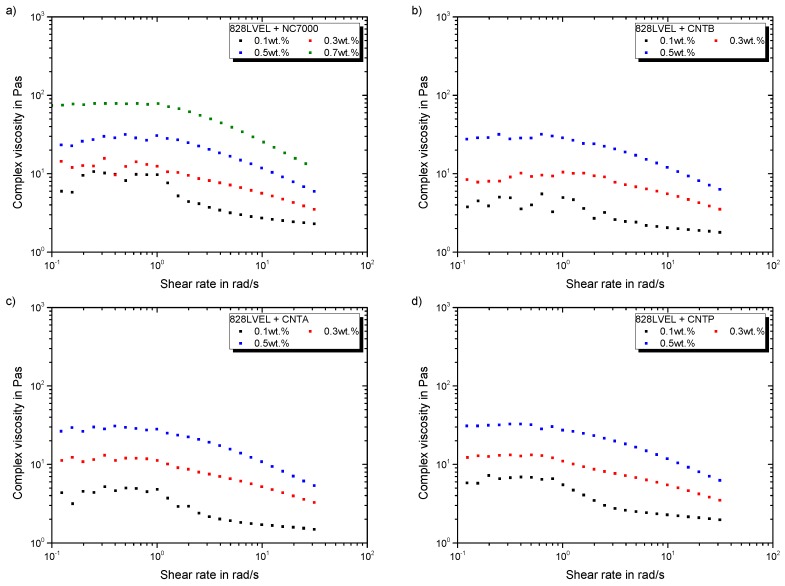
Rheological behavior of MWCNT modified 828LVEL and RIMH137/134 (**a**) NC7000 (**b**) CNTB (**c**) CNTA (**d**) CNTP.

Figure 5a–d give the rheological behavior of investigated MWCNT composites, except CNTN. All MWCNT modified epoxy systems show a similar increase of viscosity with increasing filler content. Furthermore, the critical shear rate for beginning of shear-thinning is not affected by the filler loading. All used MWCNT offer a specific surface area of about 300 m^2^/g (compare Table 2) leading to the same rheological behavior.

**Figure 6:**
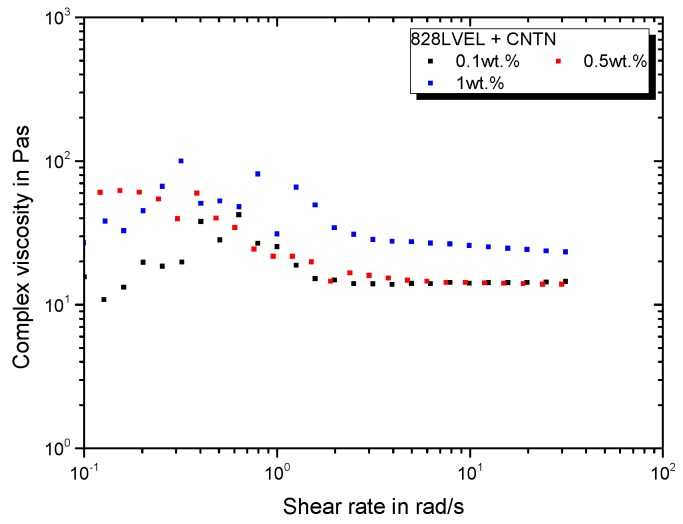
Rheological behavior of CNTN modified 828LVEL and RIMH137/134.

Figure 6 gives the rheological behavior for the CNTN modification. Due to the elastomeric and amino-functionalized side chains, the viscosity rises distinctively with increasing filler loading of CNTN. It is important to note that the filler loading of CNTN relates to the pure CNT content without elastomer. After shear-thinning the complex viscosity reaches a plateau, which is much higher compared to pristine MWCNT’s viscosity.

**Figure 7:**
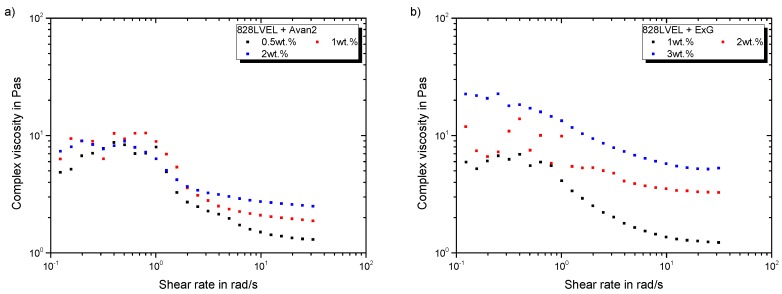
Rheological behavior of Graphene modified 828LVEL and RIMH137/134 (**a**) FLG (**b**) ExG.

Figure 7a gives the development of complex viscosity with increasing shear rate for FLG and Figure 7b for ExG modified epoxy resin. For planar FLG and ExG, the increase in complex viscosity in the low shear rate region is one magnitude lower compared to MWCNT, even up to a filler loading of 3 wt.%. A previous study revealed a strong exfoliation for this particle type leading to a very good wetting of the layers, which simplifies sliding of them [28] and eases the dispersion process. The good wettability results in low viscosities of the composite. All curves for FLG differ only in the high shear rate range. Higher filler loading lead to increased plateau viscosities after shear-thinning. ExG increases the viscosity in the low shear rate region with a filler loading of 3 wt.%. Below this filler loading the viscosity is not increased in the low shear rate region. After shear-thinning the viscosity is higher for increased filler loading.

**Figure 8:**
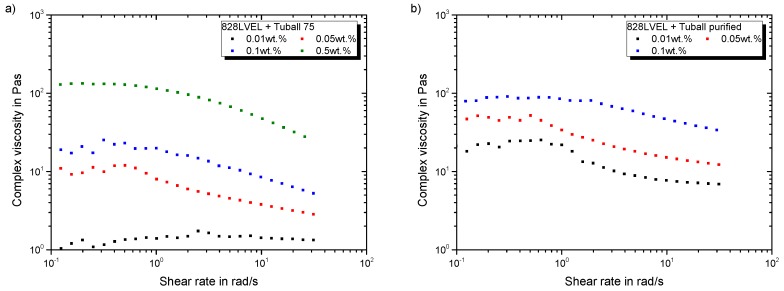
Rheological behavior of SWCNT modified 828LVEL and RIMH137/134 (**a**) Tuball 75 (**b**) Tuball purified.

Figure 8a gives the rheological behavior of Tuball 75 and Figure 8b the purified SWCNT, respectively. An addition of 0.01 wt.% of Tuball 75 does not affect the rheological behavior. Starting with a filler loading of 0.05 wt.% the typical plateau arises and the viscosity increases. The purified Tuball SWCNT exhibit the highest impact on the viscosity. An addition of 0.01 wt.% of purified SWCNT results in the same rheological behavior as for an addition of 0.1 wt.% Tuball 75. This relates to their extensive specific surface area and high purity. Therefore, they possess a higher carbon content per weight, compared to Tuball 75.

**Figure 9:**
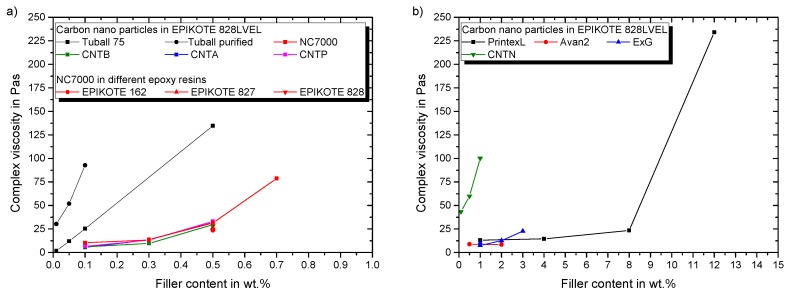
Impact of particle modification on the rheological properties for (**a**) evaluated MWCNT and SWCNT in epoxy resin with curing agent (**b**) evaluated planar particles, CB and CNTN in epoxy resin with curing agent.

Figure 9a sums up the change of viscosity in dependency of the filler loading of used SWCNT and MWCNT as well as the effect of different molecular weight epoxy resins. Only the resin’s molecular weight influences the viscosity of the composite for same particle type and filler loading, despite the way of plasma treatment and supplier. This is lower for lower molecular weight resins and is particularly pronounced for Epikote 162. The increase in viscosity is mainly dependent on the offered specific surface area. Figure 9b shows the impact of CB, CNTN, and all planar particles on the viscosity. In conclusion, particles with lower specific surface area allow good composite processing.

### 3.2. Conductivity

**Figure 10:**
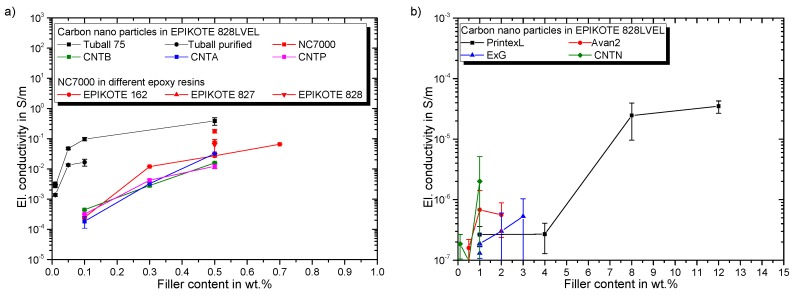
Electrical percolation behavior of (**a**) evaluated MWCNT and SWCNT in epoxy resin (**b**) evaluated planar particles, CB and CNTN in epoxy resin.

Figure 10a gives the electrical percolation behavior in dependency of SWCNT and MWCNT filler content. The SWCNT show an electrical conductivity of 10^−3^ S/m, even at low filler loading of 0.01 wt.%. Results for Tuball 75 are in good accordance with reported values in [10]. The purified SWCNT modification results in lower final conductivities compared to Tuball 75. A ternary network is formed for the non-purified variant, due to the metallic impurities, leading to improved network formation. This effect is reported by Sumfleth et al. [39], as well. All MWCNT composites show a similar percolation behavior, independent of their functionalization. Due to lower resin viscosity the agglomeration during curing is more pronounced for Epikote 162 resulting in higher conductivities. Martin et al. reported this correlation as well [40]. The electrical conductivity of carbon nanoparticle-polymer composites is dominated by electron hopping and formation of conductive networks, while network formation is the governing mechanism after percolation [41]. Seidel et al. revealed that electron hopping is much more pronounced for SWCNT compared to MWCNT, leading to much lower percolation thresholds [42]. Figure 10b gives the percolation behavior of CNTN, CB, and planar particles. Despite the high filler loading of CNTN the conductivity is much lower in comparison to other MWCNT. This can be explained by the elastomeric side chains covering the CNTs and thus hindering the build-up of a conductive particle network. CB percolates between 4 and 8 wt.%, which is also reported in a prior study for a comparable CB type [9]. Both investigated planar particles have a low impact on the electrical conductivity even up to filler loadings of 3 wt.%. The significant increase in electrical conductivity (electrical percolation) arises for all particles at lower filler loadings compared to the corresponding filler loading, which results in viscosity increase (rheological percolation). The explanation is given by the different network formation mechanisms. The tunnel distance mainly influences the electrical conductivity, whereas for an increase in viscosity a strong physical interaction of filler particles network is required to ensure a stiff network [43].

### 3.3. DMTA

**Figure 11:**
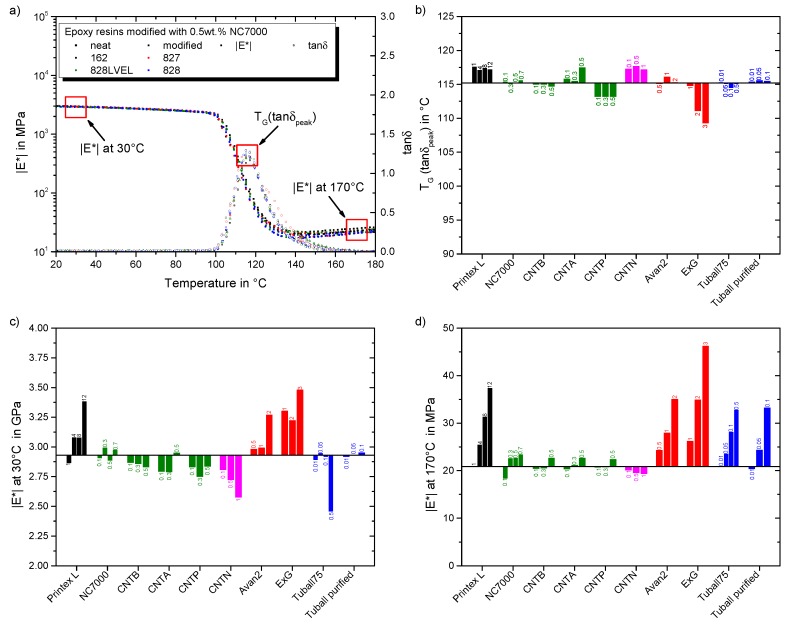
Resulting DMTA properties: (**a**) impact of 0.5 wt.% NC7000 in epoxy resins Epikote 162, 827, 828LVEL, and 828 (**b**) effect of evaluated particles on the glass transition temperature (**c**) effect of evaluated particles on the complex modulus at 30 °C (**d**) effect of evaluated particles on the complex modulus at 170 °C.

The influence of particle type and filler loading as well as molecular weight of resin on dynamic-thermo-mechanical properties are discussed in this section. Below the glass transition temperature $T_{G}$ a high modulus is desirable. For temperatures exceeding $T_{G}$ the complex modulus reveals the strength of the particle network interconnection. Figure 11a gives the resulting DMTA curves for all investigated resins and their modification with 0.5 wt.% NC7000 MWCNT. At low temperatures, the complex modulus is neither affected by the particles nor by the molecular weight of the resins. A shift of the glass transition temperature $T_{G}$, evaluated at maximum of damping factor tanδ, is also not observed. In the high temperature regime (exceeding $T_{G}$) the complex modulus increases with decreasing molecular weight of the resins. A modification with NC7000 increases the complex modulus at high temperatures, too. This indicates the formation of a percolated particle network and correlates with the previously observed increase in viscosity. For further investigations, the complex modulus is evaluated for all composites at 30 and 170 °C. The shift in $T_{G}$ is analyzed by the peak temperature of the damping factor $T(tan\delta_{peak})$.

Figure 11b gives the influence of particle type and filler loading on the $T_{G}$. The black solid line indicates the value for the neat Epikote 828LVEL. CB increases the $T_{G}$ independently of the filler loading. NC7000 and CNTB do not influence the $T_{G}$. Beginning with rheological percolation, the $T_{G}$ rises for CNTA. For CNTP, $T_{G}$ is lower compared to the neat resin. Argon plasma treatment does not only improve the electrical percolation but also leads to a slight improvement of the $T_{G}$, while atmospheric plasma treatment lowers electrical conductivity and $T_{G}$ of the composite. The amino-functionalized elastomeric side chains of CNTN increase the $T_{G}$ prospectively due to an increased cross-linking. FLG has no influence on the resulting $T_{G}$. ExG decreases the $T_{G}$ for filler loadings over 1 wt.%. This is in correspondence to the observed viscosity increase. Prolongo et al. reported that graphene sheets hinder the curing and lower cross-linking density [44]. This results in a higher mobility of the polymer chains and thus lower $T_{G}$. Figure 11c gives an overview on the development of the complex modulus in the enthalpy elastic state for the manufactured composites. The black solid line indicates the value for the neat Epikote 828LVEL. For CB, the complex modulus increases with increasing filler loading. A filler loading of 12 wt.% leads to the highest increase. NC7000 shows no effect on complex modulus at this temperature. For CNTB the modulus decreases with increasing filler loading. For plasma treated CNT the complex modulus decreases until 0.3 wt.% and then increases for a filler loading of 0.5 wt.%. This increase correlates with the rheological percolation. For CNTN the complex modulus drops with increasing filler loading, resulting from the high elastomeric content. For planar FLG and ExG the complex modulus increases with increasing filler loading. SWCNT do not affect complex modulus below a filler loading of 0.5 wt.%. A significant decrease is observed for 0.5 wt.% filler loading of Tuball 75. This attributes to the dominating network of flexible SWCNT within the composite. Figure 11d shows the dependency of complex modulus in the entropy elastic state onparticle type and filler loading. The black solid line indicates the value for the neat Epikote 828LVEL. With increasing filler loading of CB the complex modulus rises. For all MWCNT types the complex modulus increases with beginning of rheological percolation. Due to the elastomeric chains the complex modulus drops with increasing filler loading of CNTN. Increased filler loading of FLG, ExG, and both SWCNT types significantly increases the complex modulus.

### 3.4. Fracture Toughness Mode I and II

**Figure 12:**
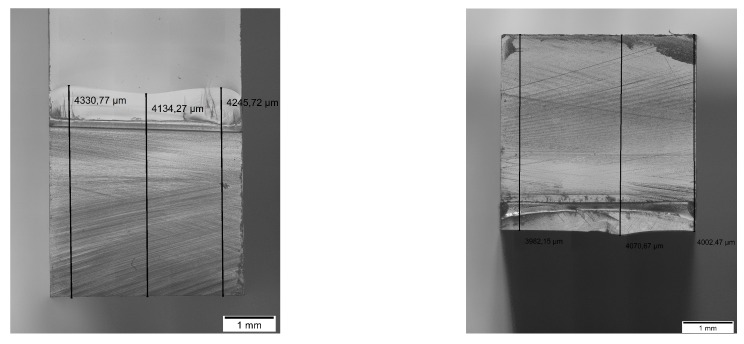
Exemplary machined notch and pre-crack for (**left**) mode I and (**right**) mode II.

Figure 12 shows exemplary the machined notch with the inserted pre-crack of the mode I (left) and mode II (right). The crack length is homogeneous and the crack tip very sharp due to the introduced notching technique.

**Figure 13:**
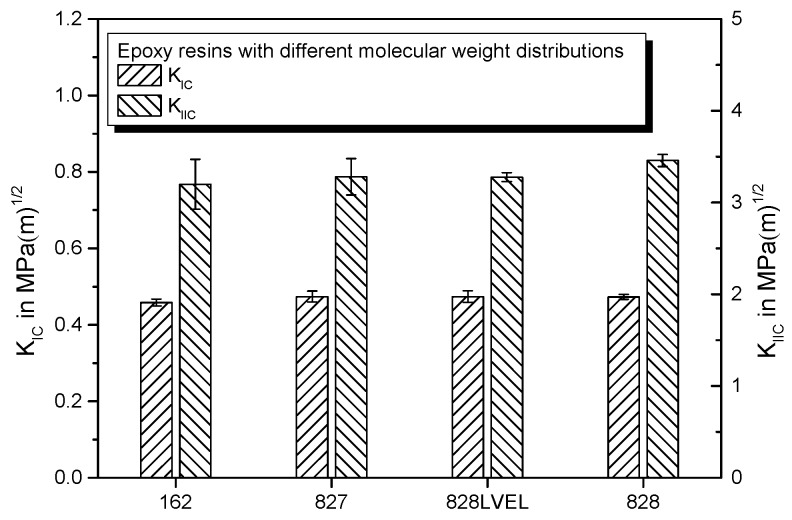
Comparison of fracture toughness in mode I and mode II for epoxy resins Epikote 162, 827, 828LVEL, and 828.

Figure 13 gives the stress intensity factors $K_{IC}$ and $K_{IIC}$ for the different molecular weight epoxy resins. The $K_{IC}$ value is lowest for Epikote 162, which offers the lowest molecular weight. Highest $K_{IIC}$ values are achieved with Epikote 828, which offers the highest molecular weight. The values are in a similar range as reported by Liu et al. [45].

#### 3.4.1. Mode I

For mode I fracture the reinforcement mechanisms are crack separation, bifurcation, pinning, deflection and separation of particles [17,18]. The results of the influence of nanoparticle type and filler loading on the mode I fracture toughness are summarized by Figure 12. CB shows a maximum at 8 wt.% filler loading (Figure 12a).

**Figure 14:**
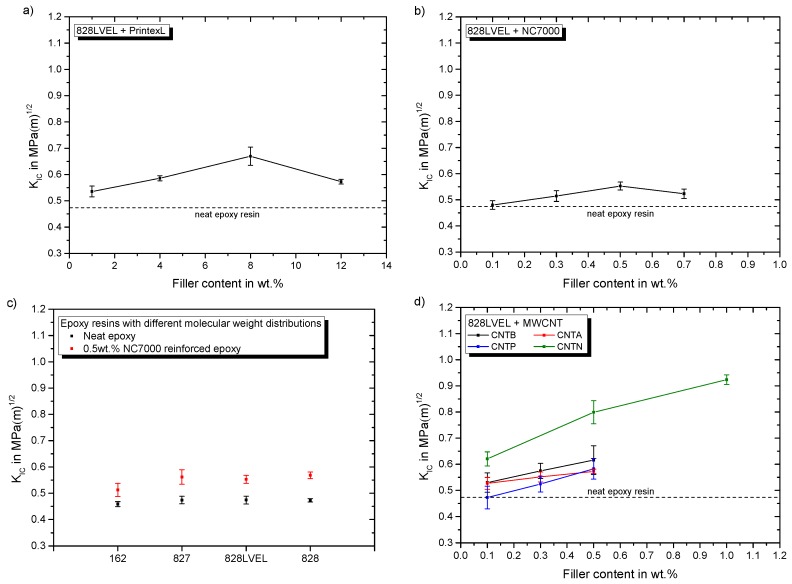

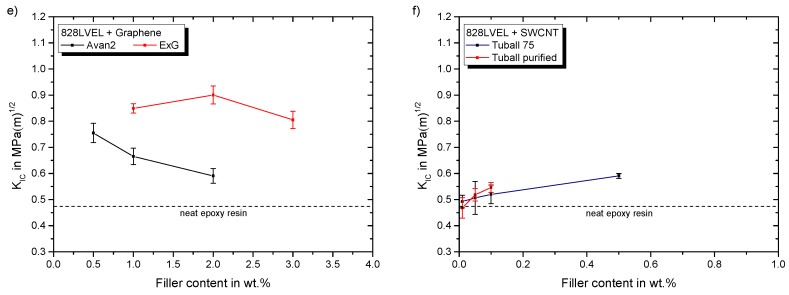
Particles impact on fracture toughness in mode I for (**a**) CB in Epikote 828LVEL (**b**) NC7000 in Epikote 828LVEL (**c**) 0.5 wt.% NC7000 in Epikote 162, 827, 828LVEL, and 828 (**d**) CNTB, CNTA, CNTP and CNTN in Epikote 828LVEL (**e**) Avan2 and ExG in Epikote 828LVEL (**f**) Tuball 75 and Tuball purified in Epikote 828LVEL.

NC7000 exhibit a maximum of the $K_{IC}$, too (Figure 12b). Both maxima lay in the range of the rheological percolation, which is also reported by Nadiv et al. [14,15]. Figure 14c shows the impact of 0.5 wt.% NC7000 on the $K_{IC}$ of the different molecular weight resins. The increase in fracture toughness in mode I is lowest for Epikote 162. Figure 14d gives the impact of atmospheric and argon plasma functionalization on the fracture toughness in mode I in dependency of their filler loading. Furthermore, the influence of CNTN is shown. CNTN show the highest potential for fracture toughness improvement with nanotubes, whereby the plasma treatments have minor influence on the mode I fracture toughness. Figure 14e shows the influence of the used planar particles on the fracture toughness. The ExG modification results in a maximum at 2 wt.% filler loading, similarly high as CNTN at 1 wt.%. For FLG the maximum in mode I fracture toughness supposedly lays below 0.5 wt.%. A maximum for this particle type is reported at 0.05 wt.% [46]. A correlation with the rheological or electrical percolation for planar particles is not possible. Figure 14f gives similar improvement in fracture toughness for both SWCNT types. They result in lower enhancement due to their flexible nature. For pristine carbon nanotubes the best improvement is achieved with 0.5 wt.% of CNTB.

#### 3.4.2. Mode II

**Figure 15:**
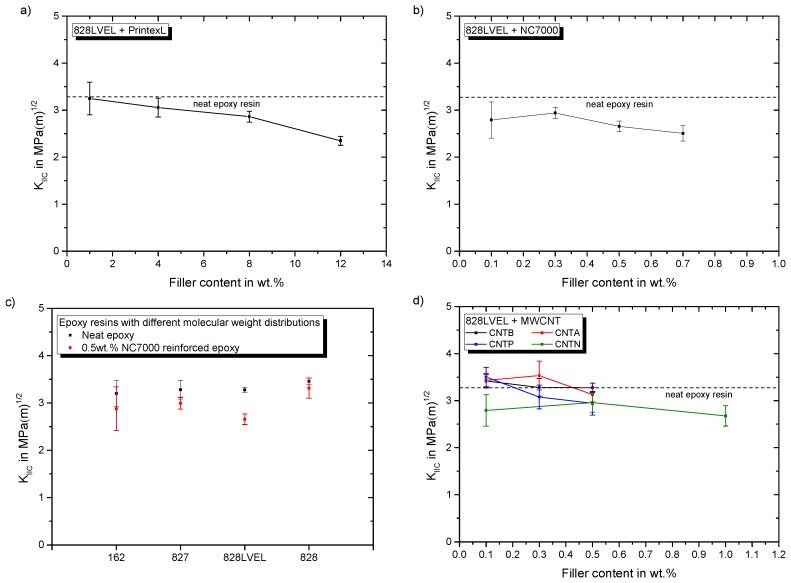

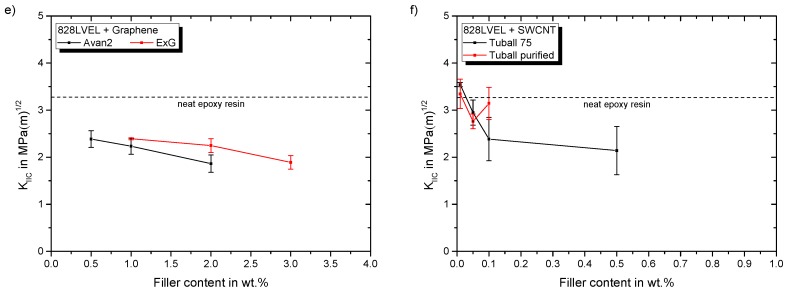
Particles impact on fracture toughness in mode II for (**a**) CB in Epikote 828LVEL (**b**) NC7000 in Epikote 828LVEL (**c**) 0.5 wt.% NC7000 in Epikote 162, 827, 828LVEL, and 828 (**d**) CNTB, CNTA, CNTP and CNTN in Epikote 828LVEL (**e**) Avan2 and ExG in Epikote 828LVEL (**f**) Tuball 75 and Tuball purified in Epikote 828LVEL.

Figure 15a,b shows the impact of CB and NC7000 filler content on the fracture toughness in mode II, respectively.

**Figure 16:**
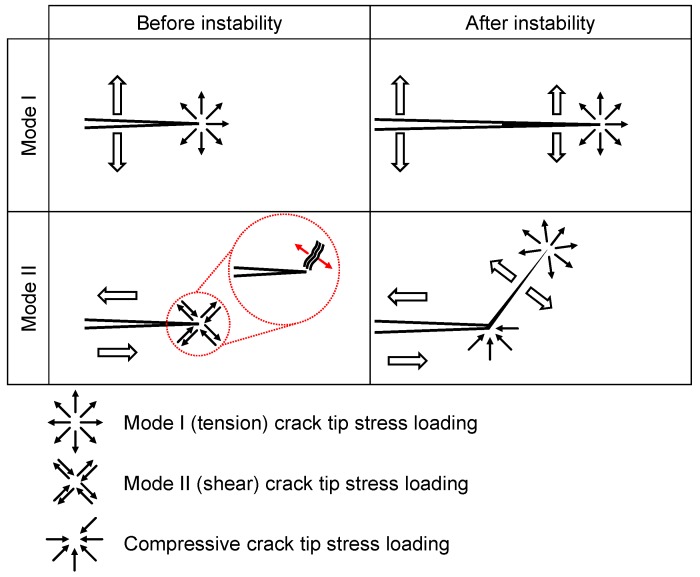
Illustration of crack propagation before and after instability in (**top**) mode I and (**bottom**) mode II with craze initiation at particle adapted from [32].

For both particle types the $K_{IIC}$ value drops with increasing filler loading. The influence of 0.5 wt.% NC7000 on the evaluated epoxy resins is given in Figure 15c. For all resins $K_{IIC}$ decreases with this particle modification. This is less pronounced for the highest molecular weight resin Epikote 828. CNTB and CNTA do not weaken the fracture toughness in mode II, as shown in Figure 15d. Atmospheric plasma treated CNTP, offering a higher oxygen content, decrease the fracture toughness in mode II with increasing filler content. The $K_{IIC}$ value drops for a CNTN modification to a constant lower value, seemingly independent of the filler concentration. Figure 15e gives the influence of used planar particles on the fracture toughness. The fracture toughness is reduced with increasing filler loading. The influence of filler loading of SWCNT on $K_{IIC}$ is shown in Figure 15f. With increasing filler content of Tuball 75, $K_{IIC}$ decreases. This reduction is less pronounced for the purified SWCNT at the investigated range of filler contents. Surprisingly, only for low filler contents of CNTB, CNTA, and CNTP a slight increase of $K_{IIC}$ is observed, whereas CNTA modification shows the highest potential. The measurement of $K_{IIC}$ is discussed controversially in literature, because it is highly dependent on notch shape and loading condition [47]. Many studies report an improvement of fracture toughness in mode II due to nanoparticle modification e.g., [19,20,23], but the mechanisms are not elaborated comprehensively. It is well known that cracks in mode II try to initiate from the crack tip in the direction of the inserted pre-crack and take onwards a curved path converging the direction of mode I loading, under an angle of 70° from the initial direction [48]. Ramsteiner states in his publication that the crack often initiates in mode I direction, even for mode II loading due to a non-ideal crack tip. Therefore, $K_{IIC}$ is often reported to be in the range of $K_{IC}$, due to inaccurate notch preparation [48]. Our sharp pre-crack prevents crack initiation in mode I in shear loading. The process zone in mode II is three times larger compared to mode I, as already shown in Figure 2. Hence, more energy can be stored in the plastic zone in mode II, leading to higher fracture toughness for shear loading of neat polymers. Figure 16 schematically shows the loading conditions for mode I and mode II at the crack tip before and after crack propagation.

**Figure 17:**
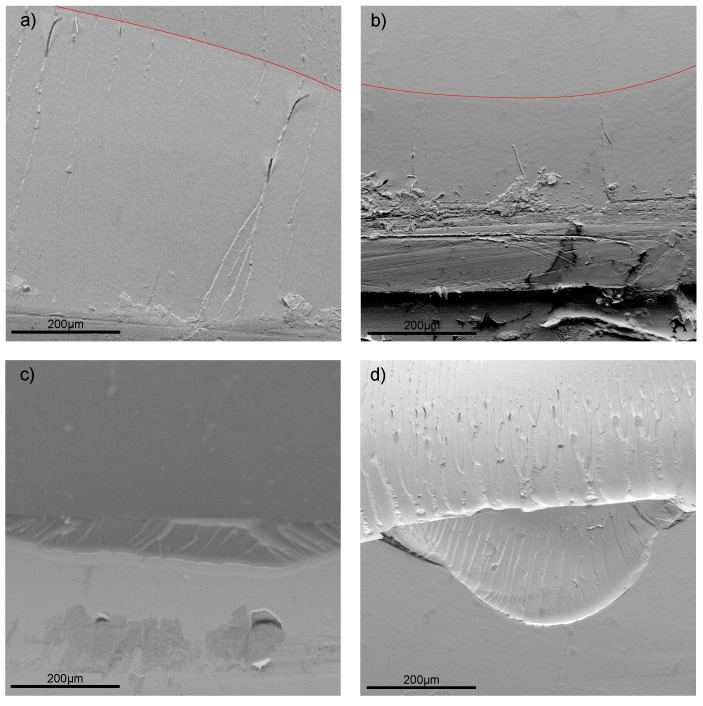
SEM images of the fracture surfaces of 0.1 wt.% SWCNT in Epikote 828LVEL (**a**) Tuball purified mode I (**b**) Tuball 75 mode II (**c**) Tuball purified mode II (**d**) Tuball 75 mode II.

In mode I the crack propagation initiates in the direction of the pre-crack and propagates further in this direction due to crack opening in tension mode. Figure 17a,b show the SEM images of the with 0.1 wt.% Tuball purified and Tuball 75 modified composite in mode I, respectively. The red line indicates the pre-notch. For both modifications, the crack propagates in the direction of the pre-notch.

For mode II the crack tries to initiate, as already stated, as well in the direction of the pre-crack. However, the propagation is in mode I under an angle of 70°, due to the lower fracture toughness in mode I. For particle filled polymers the crack may initiate in shear loading in mode I, caused by initiation of craze formation at particles. Crazing in polymers is initiated at defects or impurities [49,50]. Zhang et al. made this observation for nano composites, too [51]. Craze formation is more pronounced for lager particles and more likely for higher filler loading. The weakly bonded layered structure of the planar particles further promotes crack initiation in shear loading condition. Figure 17c,d show the SEM images of the 0.1 wt.% Tuball purified and Tuball 75 modified composite in mode II, respectively. For Tuball 75 modified samples the disruption at crack initiation point is much deeper and has a larger excess compared to the purified variant. This contributes to the impurities in the Tuball 75 SWCNT variant, which promote crack initiation. Particularly at the edges of the disruption, the formation of crazes is visible. This is not the case for the purified variant, supporting the mechanisms given in Figure 16.

## 4. Summary

**Figure 18:**
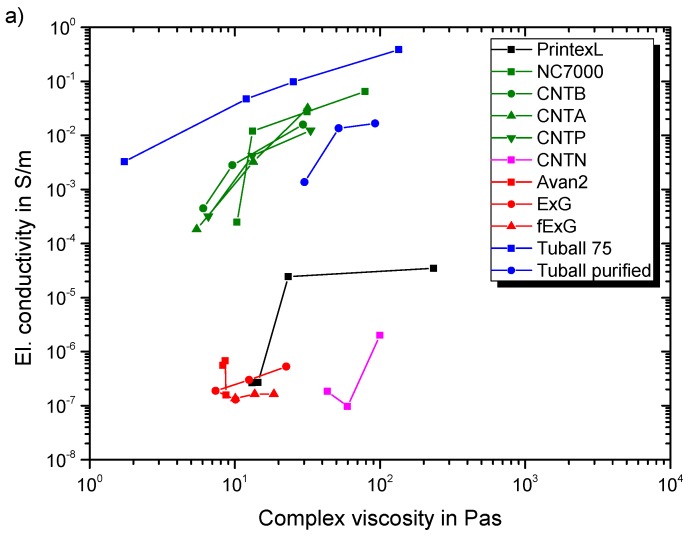
Summary of the electrical conductivity (cured state) in correspondence to the complex viscosity for all evaluated particle types in Epikote 828LVEL/RIMH 137/134 (liquid state).

Our results show highest decrease in $K_{IIC}$ for FLG, ExG, and pristine Tuball 75. Therefore, the decrease is more pronounced for SWCNT with metal impurities. CNTN feature the same elastomer domain size independent of the filler loading and therefore have the same decrease. Application demands a compromise between the enhancement of physical properties, in this case electrical conductivity and fracture toughness, and processability represented by the resulting composite’s viscosity. Figure 18 shows the electrical conductivity and the corresponding viscosities for the modified Epikote 828LVEL. Tuball 75 modification results in highest electrical conductivity at any given viscosity. Also, all types of MWCNT show good results.

**Figure 19:**
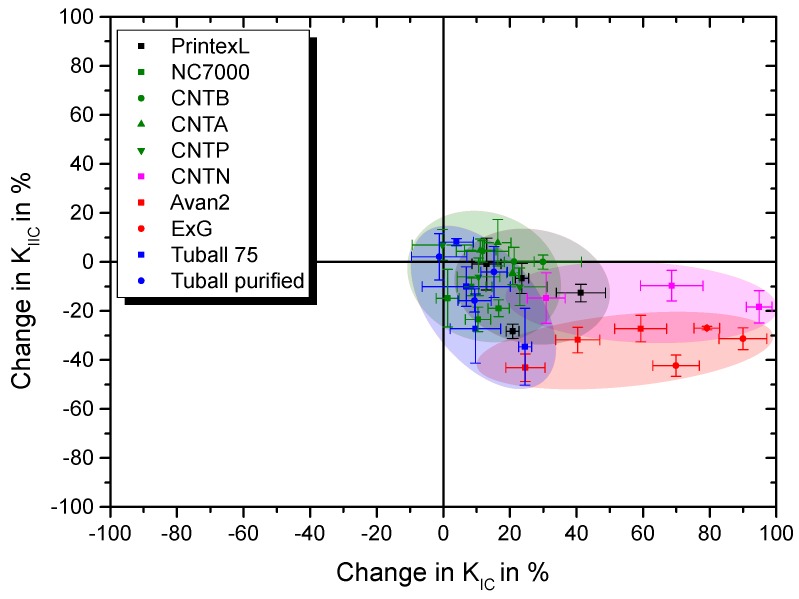
Summary of resulting $K_{IIC}$ and $K_{IC}$ values for all investigated particle types in Epikote 828LVEL.

Figure 19 gives $K_{IIC}$ and the corresponding $K_{IC}$ values for the composites. Only Tuball 75 at 0.01 wt.% filler loading and CNTA up to 0.3 wt.% increase both fracture toughness values. CNTN modification results in a low decrease in $K_{IIC}$ accompanying a high increase in $K_{IC}$, but offer a very low electrical conductivity and a comparably high viscosity increase. Therefore, Tuball 75 show highest application potential regarding requirement of electrical conductivity and ExG for improvement of mode I fracture toughness.

## 5. Conclusions

In conclusion, the novel three-step pre-cracking method allowed an exact introduction of the pre-crack and led to low variation in fracture toughness values. In particular, the measured mode II fracture toughness values were as high as reported in literature, due to prevention of crack initiation in tension mode. The plastic zone in front of the crack tip in mode II stores much more energy compared to mode I, resulting from its dimension. For mode II loading of a pre-crack in a nanocomposite, the particles act as crack initiator in tension mode, lowering the fracture toughness in mode II. SWCNT Tuball 75 offer the highest conductivities at any given viscosity and the lowest percolation threshold. This makes them favorable for applications where high conductivities are demanded. MWCNT and CB show a good compromise of processability and physical property enhancement. Addition of planar and globular carbon nanoparticles leads to comparably low electrical conductivities, but ExG can achieve a very high mode I fracture toughness at only slightly increased viscosities. All fillers lead to a decrease of $K_{IIC}$ above a certain loading threshold, due to promotion of crack initiation.

## Figures and Tables

**Table 1:** Properties of used epoxy resins.

Resin	Type	Epoxy Equivalent Weight in g/eq	Epoxy Group Content in mmol/kg	Viscosity at 25 °C in Pas	Physical State	Remarks
162	DGEBA	170–174	5744–5883	4.0–5.0	Liquid	Distilled, high purity grade
827	DGEBA	179–184	5430–5590	8.0–10.0	Liquid	Low viscosity
828LVEL	DGEBA	182–187	5340–5500	10.0–12.0	Liquid	Low hydrolyzable chlorine, low viscosity
828	DGEBA	184–190	5260–5420	12.0–14.0	Liquid	Standard unmodified bisphenol A resin

**Table 2:** Properties of used carbon nanoparticles.

Nanoparticle	BET Surface Area in m^2^/g	Layers, Walls or Bundle Size	Aspect Ratio	Shape	Diameter or Thickness in nm	TGA Impurities in wt.%	EDX Composition in wt.%
Printex L	125	amorphous	1	spherical	31±8	0.25	C (96.51), O (2.69), S (0.80)
NC7000	321	10±2	150	cylindrical	12±3	9.17	C (92.15), O (5.96), Al (1.81), Si (0.08)
CNTB	319	10±3	150	cylindrical	11±3	10.35	C (85.34), O (8.78), Al (5.07), Fe (0.36), Co (0.22), Si (0.15), S (0.08)
CNTA	296	9±2	150	cylindrical	10±1	10.64	C (88.77), O (7.97), Al (2.73), Fe (0.24), Si (0.14), S (0.07)
CNTP	312	10±2	150	cylindrical	11±3	7.84	C (85.39), O (11.89), Al (1.79), Si (0.44), S (0.25), Na (0.25)
CNTN ${}^{⋆}$	N/A	N/A	N/A	cylindrical	N/A	4.39	C (90.53), N (4.78), O (4.21), Al (0.37), Cl (0.11)
Tuball 75	605	11±3	3570	cylindrical	1.4±0.4	17.98	C (81.96), Fe (15.01), O (2.26), Si (0.44), S (0.32)
Tuball purified	598	13±3	3570	cylindrical	1.5±0.1	2.09	C (90.61), O (6.36), Si (1.74), Cl (1.03), Fe (0.26)
FLG (Avan2)	128	10±2	6250	planar	4±1	5.81	C (88.33), O (9.15), Mn (0.65), Si (0.62), S (0.35), Al (0.32), Fe (0.24), Na (0.22), Cl (0.08), Ca (0.06)
ExG ${}^{⋆⋆}$	18	47±11	525	planar	19±5	10.90	C (88.08), O (11.02), S (0.45), Cu (0.25), Si (0.12), Na (0.08)

⋆ This particle bases on CNTB and features amino-functionalized elastomer side chains and 20  to 25 wt.% CNT content. ⋆⋆ This particle type was delivered solved in ethanol and was dried before measurements.

**Table 3:** Process parameters for dispersion on three-roll mill 80E Plus.

Step	Gap_1_ in m	Gap_2_ in m	n_1_ in Rpm	n_2_ in Rpm	n_3_ in Rpm
1	120	40	50	150	450
2	40	13	50	150	450
3–7	13	5	50	150	450

**Table 4:** Required dimensions of mode I specimens.

*B*	*W*	a=0.5W	L=5a=5d	d=0.5W
3.9 mm	7.8 mm	3.9 mm	19.5 mm	3.9 mm

**Table 5:** Required dimensions of mode II specimens.

B=0.5W	*W*	a=0.5W	L=2W	$L_{\mathrm{support}}=4W$
3.9 mm	7.8 mm	3.9 mm	15.6 mm	31.2 mm

**Figure A1:**
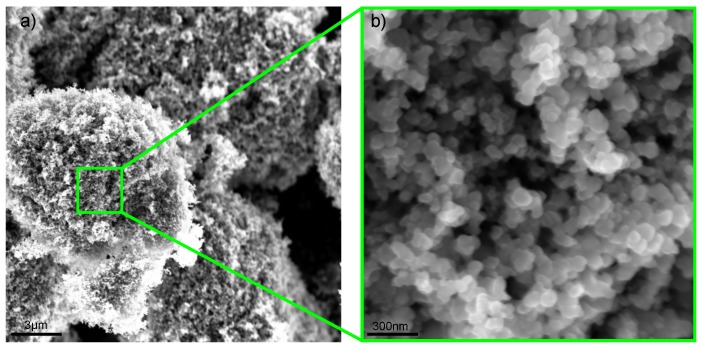
SEM images for PrintexL at magnifications of (**a**) 10 k and (**b**) 150 k.

**Figure A2:**
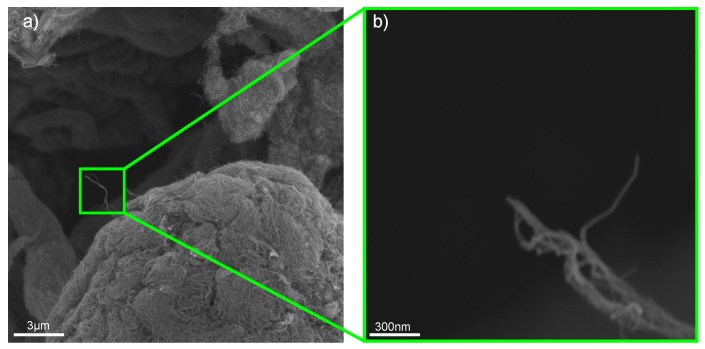
SEM images for NC7000 at magnifications of (**a**) 10 k and (**b**) 150 k.

**Figure A3:**
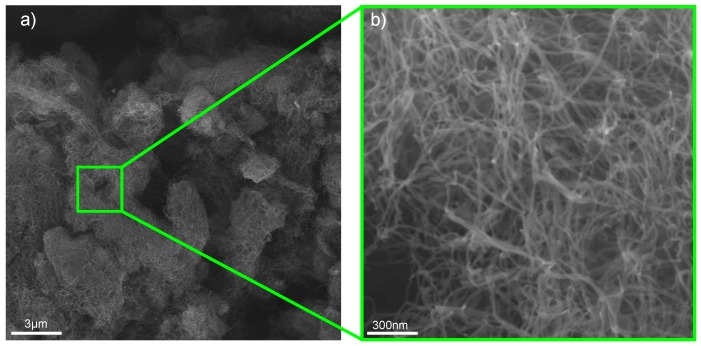
SEM images for CNTB at magnifications of (**a**) 10 k and (**b**) 150 k.

**Figure A4:**
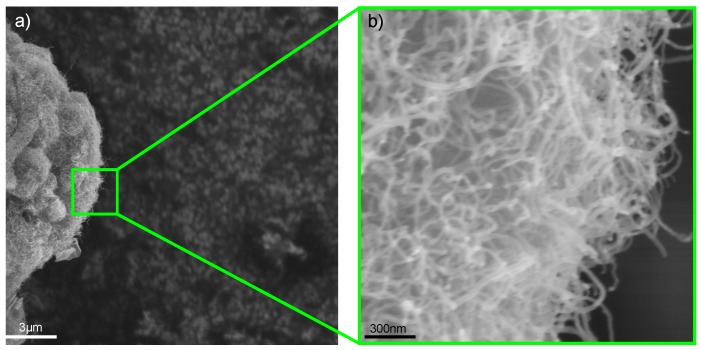
SEM images for CNTA at magnifications of (**a**) 10 k and (**b**) 150 k.

**Figure A5:**
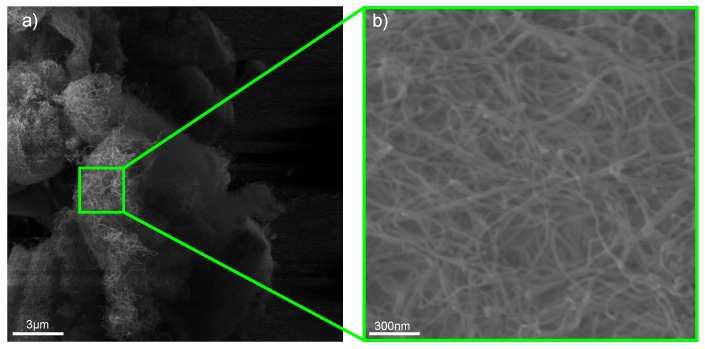
SEM images for CNTP at magnifications of (**a**) 10 k and (**b**) 150 k.

**Figure A6:**
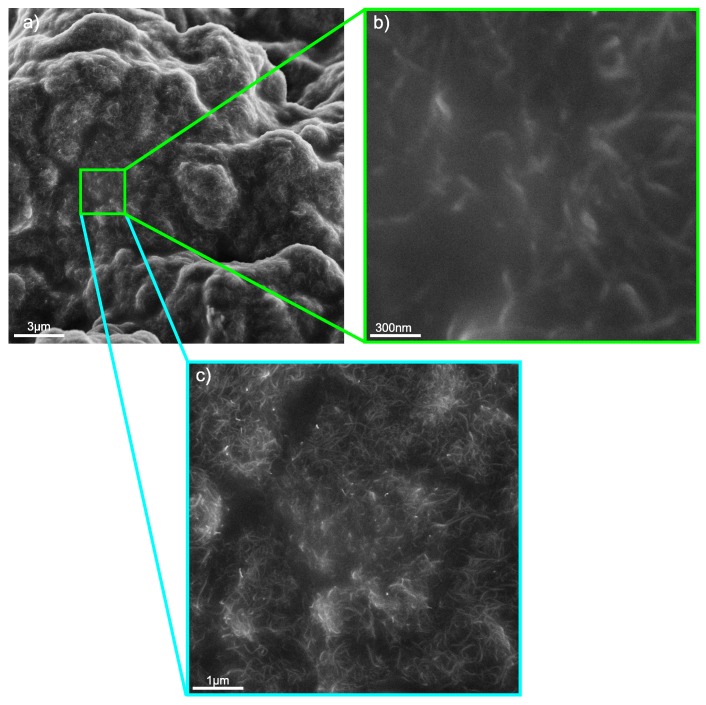
SEM images for CNTN at magnifications of (**a**) 10 k, (**b**) 150 k and (**c**) 25 k.

**Figure A7:**
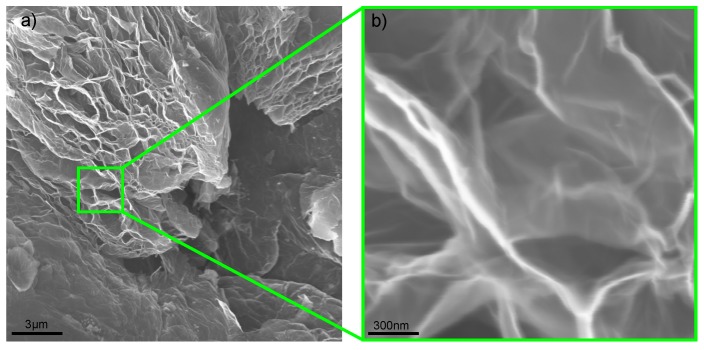
SEM images for FLG Avan2 at magnifications of (**a**) 10 k and (**b**) 150 k.

**Figure A8:**
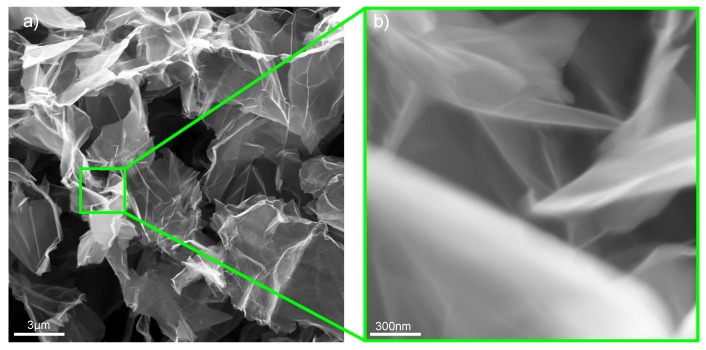
SEM images for ExG at magnifications of (**a**) 10 k and (**b**) 150 k.

**Figure A9:**
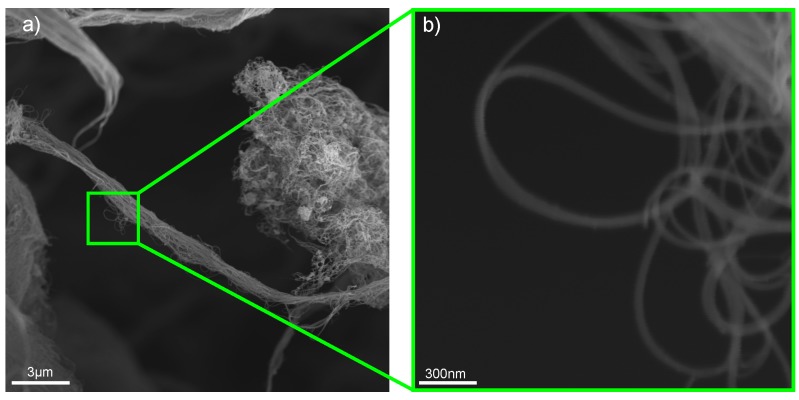
SEM images for Tuball 75 at magnifications of (**a**) 10 k and (**b**) 150 k [27].

**Figure A10:**
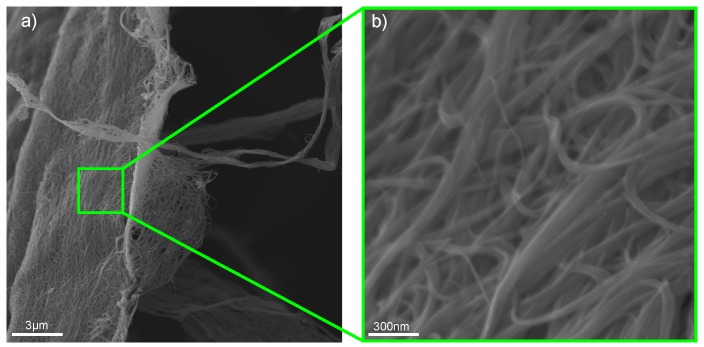
SEM images for Tuball purified at magnifications of (**a**) 10 k and (**b**) 150 k.

**Figure A11:**
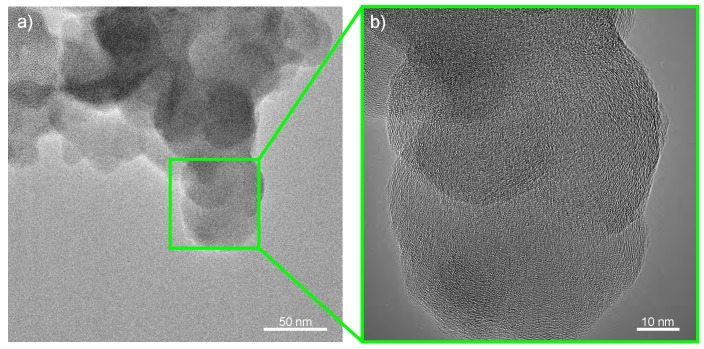
TEM images for PrintexL at (**a**) low magnification and (**b**) high magnification.

**Figure A12:**
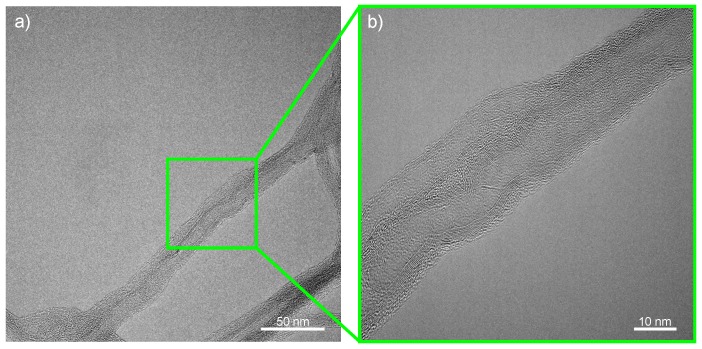
TEM images for NC7000 at (**a**) low magnification and (**b**) high magnification.

**Figure A13:**
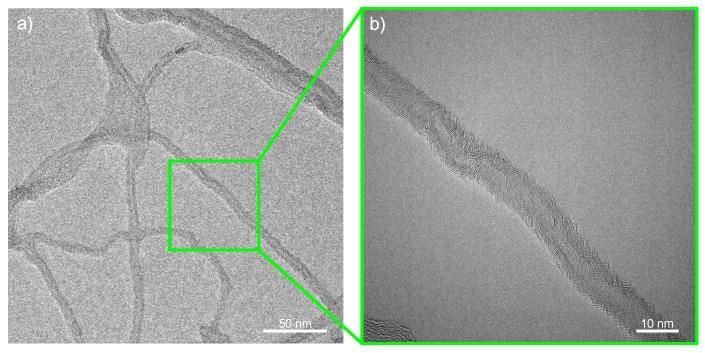
TEM images for CNTB at (**a**) low magnification and (**b**) high magnification.

**Figure A14:**
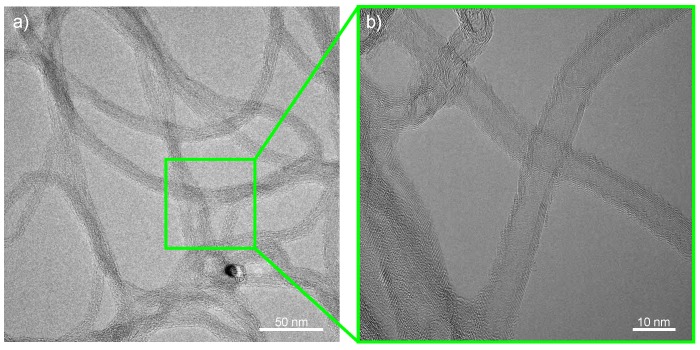
TEM images for CNTA at (**a**) low magnification and (**b**) high magnification.

**Figure A15:**
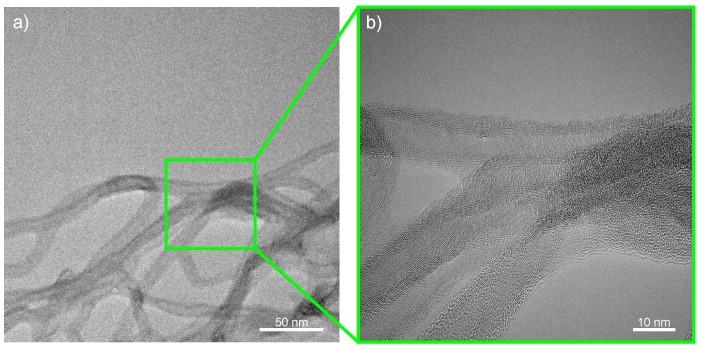
TEM images for CNTP at (**a**) low magnification and (**b**) high magnification.

**Figure A16:**
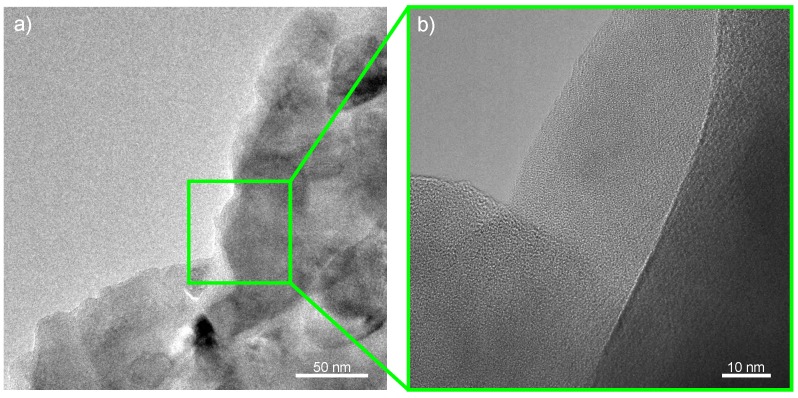
TEM images for CNTN at (**a**) low magnification and (**b**) high magnification.

**Figure A17:**
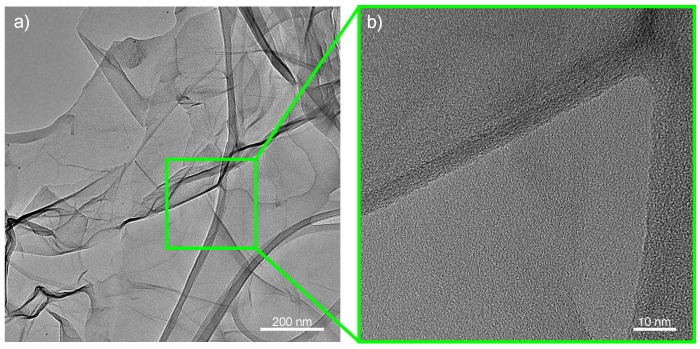
TEM images for Avan2 at (**a**) low magnification and (**b**) high magnification.

**Figure A18:**
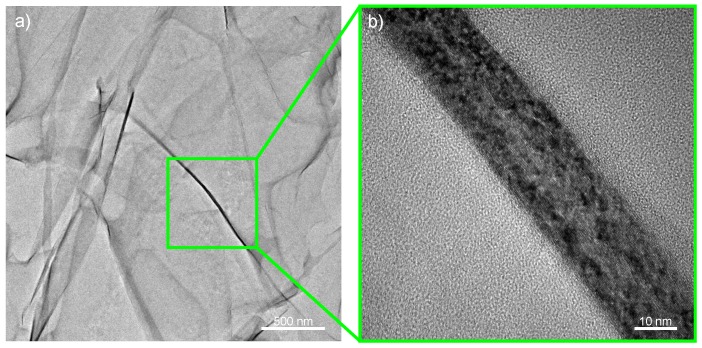
TEM images for ExG at (**a**) low magnification and (**b**) high magnification.

**Figure A19:**
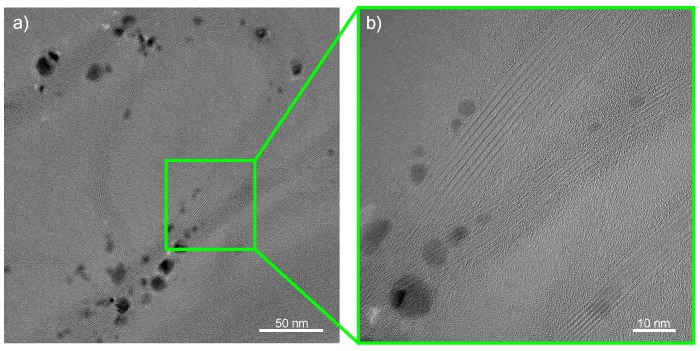
TEM images for Tuball 75 at (**a**) low magnification and (**b**) high magnification [27].

**Figure A20:**
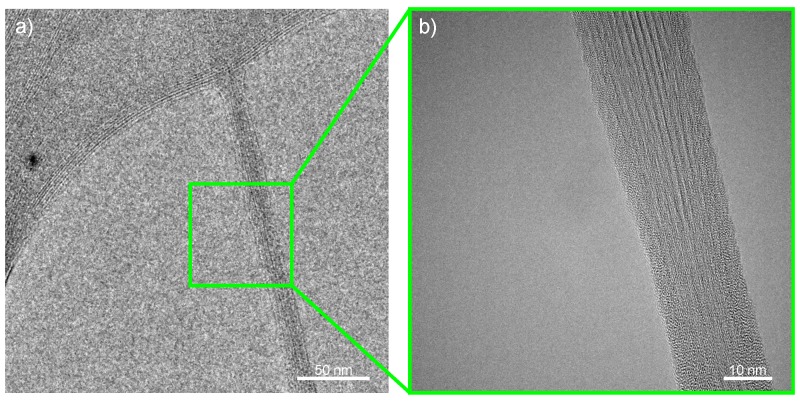
TEM images for Tuball purified at (**a**) low magnification and (**b**) high magnification.

## Abbreviations

The following abbreviations are used in this manuscript:

3PBT	three-point bending test
4PBT	four-point bending test
BET	Brunauer–Emmett–Teller theory
CB	carbon black
CNT	carbon nanotube
CNTA	argon plasma treated CNTB
CNTB	reference MWCNT
CNTN	CNTB equipped with elastomer side chains, which are amino-functionalized
CNTP	atmospheric plasma treated CNTB
ExG	electrochemically expanded graphite
FLG	few-layer graphene
MWCNT	multi-walled carbon nanotube
SEM	Scanning electron microscopy
SENB	single-edge notched bending
SWCNT	single-walled carbon nanotube
TEM	Transmission electron microscopy

## Appendix A. Morphological Characterization of Used Nanoparticles

### Appendix A.1. Scanning Electron Microscopy

To evaluate the morphology of used particles SEM images were taken at magnifications of 10 k and 150 k, respectively. Figure A1 gives the SEM images of CB PrintexL. They inhibit a globular form, which is typical for carbon blacks. The images reveal agglomerates with a diameter of 10 µm.

Figure A2 gives the SEM images of MWCNT NC7000. They inhibit a cylindrical geometry. The MWCNT are highly entangled within the agglomerates.

Figure A3, Figure A4 and Figure A5 give the SEM images of MWCNT CNTB, CNTA, and CNTP, respectively. All MWCNT from Future Carbon inhibit the same cylindrical geometry. In comparison to NC7000 the network within the agglomerates appears to be less dense.

Figure A6 gives the SEM images of CNTN. The MWCNT are equipped with amino-functionalized elastomer side chains and are well incorporated in the elastomer. Additionally, Figure A6c shows CNTN at a magnification of 25 k and reveals the distribution of the MWCNT.

Figure A7 and Figure A8 give the SEM images of planar FLG and ExG, respectively. FLG Avan2 appears in a higher agglomerated state compared to ExG.

Figure A9 and Figure A10 give the SEM images of SWCNT Tuball 75 and Tuball purified, respectively. Both appear in a highly agglomerated and entangled state. The purified SWCNT offer low metallic impurity content.

### Appendix A.2. Transmission Electron Microscopy

To evaluate the morphology of used particles TEM images were taken at two magnifications. Figure A11 gives the TEM images of CB PrintexL. They show an amorphous structure.

Figure A12, Figure A13, Figure A14 and Figure A15 give the TEM images of MWCNT NC7000, CNTB, CNTA, and CNTP, respectively. They all show a similar appearance.

Figure A16 gives the TEM images of CNTN. The elastomer is clearly visible and the MWCNT are well incorporated into the polymer.

Figure A17 and Figure A18 give the TEM images of planar FLG and ExG, respectively. FLG Avan2 has a less graphene layers compared to ExG.

Figure A19 and Figure A20 give the TEM images of SWCNT Tuball 75 and Tuball purified, respectively. Both appear in a highly bundled state. The purified SWCNT offer low metallic impurity content.

## References

[1] Müller K., Bugnicourt E., Latorre M., Jorda M., Echegoyen Sanz Y., Lagaron J.M., Miesbauer O., Bianchin A., Hankin S., Bölz U. (2017). Review on the Processing and Properties of Polymer Nanocomposites and Nanocoatings and Their Applications in the Packaging, Automotive and Solar Energy Fields. Nanomaterials.

[2] Jin F.L., Li X., Park S.J. (2015). Synthesis and application of epoxy resins: A review. J. Ind. Eng. Chem..

[3] Haward R.N., Young R.J. (2012). Physics of Glassy Polymers.

[4] Domun N., Paton K.R., Hadavinia H., Sainsbury T., Zhang T., Mohamud H. (2017). Enhancement of Fracture Toughness of Epoxy Nanocomposites by Combining Nanotubes and Nanosheets as Fillers. Materials.

[5] Domun N., Hadavinia H., Zhang T., Sainsbury T., Liaghat G.H., Vahid S. (2015). Improving the fracture toughness and the strength of epoxy using nanomaterials—A review of the current status. Nanoscale.

[6] Shtein M., Nadiv R., Lachman N., Daniel Wagner H., Regev O. (2013). Fracture behavior of nanotube–polymer composites: Insights on surface roughness and failure mechanism. Compos. Sci. Technol..

[7] Nadiv R., Vasilyev G., Shtein M., Peled A., Zussman E., Regev O. (2016). The multiple roles of a dispersant in nanocomposite systems. Compos. Sci. Technol..

[8] Deng F., Zheng Q.S. (2008). An analytical model of effective electrical conductivity of carbon nanotube composites. Appl. Phys. Lett..

[9] Meeuw H., Viets C., Liebig W.V., Schulte K., Fiedler B. (2016). Morphological influence of carbon nanofillers on the piezoresistive response of carbon nanoparticle/epoxy composites under mechanical load. Eur. Polym. J..

[10] Meeuw H., Radek M., Fiedler B. (2019). Development of a colored GFRP with antistatic properties. AIP Conf. Proc..

[11] Fan Z., Advani S.G. (2007). Rheology of multiwall carbon nanotube suspensions. J. Rheol..

[12] Rahatekar S.S., Koziol K.K.K., Butler S.A., Elliott J.A., Shaffer M.S.P., Mackley M.R., Windle A.H. (2006). Optical microstructure and viscosity enhancement for an epoxy resin matrix containing multiwall carbon nanotubes. J. Rheol..

[13] Reddy C.S., Zak A., Zussman E. (2011). WS2 nanotubes embedded in PMMA nanofibers as energy absorptive material. J. Mater. Chem..

[14] Nadiv R., Shachar G., Peretz-Damari S., Varenik M., Levy I., Buzaglo M., Ruse E., Regev O. (2018). Performance of nano-carbon loaded polymer composites: Dimensionality matters. Carbon.

[15] Nadiv R., Shtein M., Shachar G., Varenik M., Regev O. (2017). Optimal nanomaterial concentration: Harnessing percolation theory to enhance polymer nanocomposite performance. Nanotechnology.

[16] Chandrasekaran S., Liebig W.V., Mecklenburg M., Fiedler B., Smazna D., Adelung R., Schulte K. (2016). Fracture, failure and compression behaviour of a 3D interconnected carbon aerogel (Aerographite) epoxy composite. Compos. Sci. Technol..

[17] Chandrasekaran S., Sato N., Tölle F., Mülhaupt R., Fiedler B., Schulte K. (2014). Fracture toughness and failure mechanism of graphene based epoxy composites. Compos. Sci. Technol..

[18] Chandrasekaran S., Seidel C., Schulte K. (2013). Preparation and characterization of graphite nano-platelet (GNP)/epoxy nano-composite: Mechanical, electrical and thermal properties. Eur. Polym. J..

[19] Ayatollahi M.R., Shadlou S., Shokrieh M.M. (2011). Fracture toughness of epoxy/multi-walled carbon nanotube nano-composites under bending and shear loading conditions. Mater. Des..

[20] Ayatollahi M.R., Shadlou S., Shokrieh M.M. (2011). Mixed mode brittle fracture in epoxy/multi-walled carbon nanotube nanocomposites. Eng. Fract. Mech..

[21] Zappalorto M., Salviato M., Quaresimin M. (2013). Mixed mode (I+II) fracture toughness of polymer nanoclay nanocomposites. Eng. Fract. Mech..

[22] Shadlou S., Alishahi E., Ayatollahi M.R. (2013). Fracture behavior of epoxy nanocomposites reinforced with different carbon nano-reinforcements. Compos. Struct..

[23] Quan D., Urdániz J.L., Ivanković A. (2018). Enhancing mode-I and mode-II fracture toughness of epoxy and carbon fibre reinforced epoxy composites using multi-walled carbon nanotubes. Mater. Des..

[24] Ahmadi-Moghadam B., Taheri F. (2014). Fracture and toughening mechanisms of GNP-based nanocomposites in modes I and II fracture. Eng. Fract. Mech..

[25] Benra J., Forero S. (2018). Epoxy resins reinforced with carbon nanotubes. Lightweight Des. Worldw..

[26] Parvez K., Wu Z.S., Li R., Liu X., Graf R., Feng X., Müllen K. (2014). Exfoliation of graphite into graphene in aqueous solutions of inorganic salts. J. Am. Chem. Soc..

[27] Meeuw H., Körbelin J., von Bernstorff D., Augustin T., Liebig W.V., Fiedler B. (2018). Smart dispersion: Validation of OCT and impedance spectroscopy as solutions for in-situ dispersion analysis of CNP/EP-composites. Materialia.

[28] Meeuw H., Wisniewski V.K., Köpke U., Nia A.S., Vázquez A.R., Lohe M., Feng X., Fiedler B. (2019). In-line monitoring of carbon nanoparticle epoxy dispersion processes: Insights into the process via next generation three roll mills and impedance spectroscopy. Prod. Eng..

[29] Meeuw H., Wisniewski V., Fiedler B. (2018). Frequency or Amplitude?—Rheo-Electrical Characterization of Carbon Nanoparticle Filled Epoxy Systems. Polymers.

[30] Fett T. (1991). Stress intensity factors for edge crack subjected to mixed mode four-point bending. Theor. Appl. Fract. Mech..

[31] Araki W., Nemoto K., Adachi T., Yamaji A. (2005). Fracture toughness for mixed mode I/II of epoxy resin. Acta Mater..

[32] Kalthoff J.F. (2003). Failure methodology of mode II loaded crack. Strength Fract. Complex..

[33] Bergmannshoff D. (2006). Das Instabilitätsverhalten Zug-/scherbeanspruchter Risse bei Variation des Belastungs-pfades. Ph.D. Dissertation.

[34] American Society for Testing and Materials (2014). Test Methods for Plane-Strain Fracture Toughness and Strain Energy Release Rate of Plastic Materials.

[35] Xiao K., Ye L., Kwok Y.S. (1998). Effects of pre-cracking methods on fracture behaviour of an Araldite-F epoxy and its rubber-modified systems. J. Mater. Sci..

[36] de Souza J.M., Yoshimura H.N., Peres F.M., Schön C.G. (2012). Effect of sample pre-cracking method and notch geometry in plane strain fracture toughness tests as applied to a PMMA resin. Polym. Test..

[37] Kuppusamy N., Tomlinson R.A. (2016). Repeatable pre-cracking preparation for fracture testing of polymeric materials. Eng. Fract. Mech..

[38] Haddadi E., Choupani N., Abbasi F. (2016). Investigation on the Effect of Different Pre-Cracking Methods on Fracture Toughness of RT-PMMA. Lat. Am. J. Solids Struct..

[39] Sumfleth J., Adroher X.C., Schulte K. (2009). Synergistic effects in network formation and electrical properties of hybrid epoxy nanocomposites containing multi-wall carbon nanotubes and carbon black. J. Mater. Sci..

[40] Martin C.A., Sandler J., Shaffer M., Schwarz M.K., Bauhofer W., Schulte K., Windle A.H. (2004). Formation of percolating networks in multi-wall carbon-nanotube—Epoxy composites. Compos. Sci. Technol..

[41] Feng C., Jiang L. (2013). Micromechanics modeling of the electrical conductivity of carbon nanotube (CNT)—Polymer nanocomposites. Compos. Part A Appl. Sci. Manuf..

[42] Seidel G.D., Lagoudas D.C. (2009). A Micromechanics Model for the Electrical Conductivity of Nanotube-Polymer Nanocomposites. J. Compos. Mater..

[43] Bauhofer W., Kovacs J.Z. (2009). A review and analysis of electrical percolation in carbon nanotube polymer composites. Compos. Sci. Technol..

[44] Prolongo M.G., Salom C., Arribas C., Sánchez-Cabezudo M., Masegosa R.M., Prolongo S.G. (2016). Influence of graphene nanoplatelets on curing and mechanical properties of graphene/epoxy nanocomposites. J. Therm. Anal. Calorim..

[45] Liu C., Huang Y., Stout M.G. (1998). Enhanced mode-II fracture toughness of an epoxy resin due to shear banding. Acta Mater..

[46] Fritsch D., Wittich H., Fiedler B. (2016). Graphene Modified CFRP for Enhanced Damage Behavior. Proceedings of the ECCM17—17th European Conference on Composite Materials.

[47] Carpinteri A., Valente S., Ferrara G., Melchiorrl G. (1993). Is mode II fracture energy a real material property?. Comput. Struct..

[48] Ramsteiner F. (1993). An approach towards understanding model II failure of poly(methyl methacrylate). Polymer.

[49] Kinloch A.J., Young R.J. (2013). Fracture Behaviour of Polymers.

[50] Dekkers M.E.J., Heikens D. (1985). Crazing and shear deformation in glass bead-filled glassy polymers. J. Mater. Sci..

[51] Zhang W., Srivastava I., Zhu Y.F., Picu C.R., Koratkar N.A. (2009). Heterogeneity in epoxy nanocomposites initiates crazing: Significant improvements in fatigue resistance and toughening. Small.

